# Radiation-before-pathology approach in the palliative oncology setting: a pragmatic clinical trial protocol (RT-NOW)

**DOI:** 10.1186/s12904-025-01724-3

**Published:** 2025-04-07

**Authors:** Sympascho Young, Melissa O’Neil, Joanna M. Laba, Timothy K. Nguyen, X. Melody Qu, Christopher D. Goodman, Glenn S. Bauman, Andrew Warner, Matthew Cecchini, David A. Palma

**Affiliations:** 1 Department of Radiation Oncology, London Health Sciences Centre, Western University, 800 Commissioners Rd. E, London, ON N6A 5W9 Canada; 2 Department of Pathology, London Health Sciences Centre, Western University, London, ON Canada

**Keywords:** Palliative radiation, Biopsy, Pragmatic trial, Empiric treatment, Medical wait times, Metastatic cancer

## Abstract

**Background:**

Patients with incurable but not-yet-biopsied cancers sometimes require urgent palliative radiation. However, wait-times for biopsy procedures and pathologic results can delay treatment, with significant consequences to patient quality of life and/or the chance of irreversible cancer complications. There is no prospective data to guide empirical decision-making in these urgent, palliative contexts.

**Methods:**

In this prospective single-arm pragmatic clinical trial, we will enrol 48 patients with incurable cancer where a biopsy is delaying urgent palliative radiation. Patients will receive empiric upfront palliative radiation without biopsy-confirmation. The primary endpoint is the rate of inappropriate radiation, defined when the patient’s biopsy shows a non-malignant entity or a malignancy that is better treated upfront with systemic therapy (or therapy other than radiation). Secondary endpoints include: histologic diagnostic accuracy, molecular testing accuracy, biopsy complications rates, evidence of radiation effect in biopsy, time from enrolment to radiation/biopsy, and Edmonton Symptom Assessment Scale (ESAS) scores. Patients are eligible only if the probability of incurable malignancy is deemed > 95% and the risk of lymphoma < 20% by the treating physician, based on clinical examination and imaging investigations.

**Discussion:**

This study will provide prospective data to guide oncologists and patients in making informed decisions when weighing the competing risks of delaying palliative radiation versus treating without pathologic confirmation.

**Trial registration:**

*ClinicalTrials.gov* Identifier: NCT06156800. Date of registration: December 5, 2023.

## Background

In palliative radiation oncology, there is often a dilemma between the urgency of starting radiation treatment versus waiting for a biopsy for definitive diagnosis. Clinicians typically prefer waiting for a biopsy to confirm a malignancy prior to radiation, due to perceived risks of incorrect management (i.e. radiating a lymphoma or non-malignant lesion) and the possibility of radiation resulting in a non-diagnostic biopsy.

With increasingly stressed healthcare systems globally, biopsy wait times have lengthened, with the consequence of significantly delaying palliative radiation, sometimes by over a month in Canada. This comes at a high cost for patients, as their disease and symptoms continue to progress during the waiting period. Wait times for treatments have been associated with patient and caregiver anxiety, depression and poor quality of life [1].

Modern decision-making for patients in a palliative context requires a more nuanced balance of competing priorities for the patient, while taking into account the new realities in a publicly-funded healthcare system. Since dose/fractionation schemes for palliative radiation usually do not depend on the specific tumour histology, in some cases, physicians will elect to deliver radiation prior to biopsy, as part of shared decision making with the patient. However, to our knowledge, this approach has not been prospectively studied. RT-NOW is a single arm pragmatic trial with the goal of prospectively assessing this approach of delivering radiation before biopsy when warranted by symptom urgency, in carefully selected patients.

There are 4 main issues that clinicians are hoping to avoid when treating without a pathologic diagnosis:

1. Creating a non-diagnostic biopsy through radiation effects on tissue.
2. Increasing the risk of biopsy complications.
3. Treating a non-cancerous process.
4. Treating a cancer that would be best managed with chemotherapy/systemic therapy as the primary treatment (i.e. lymphoma, small cell lung cancer, germ cell tumor).

### Potential issue #1: creating a non-diagnostic biopsy through radiation effects on tissue

Non-diagnostic biopsies can mean either absence of histological or molecular testing results. We estimate that the risk of histologic non-diagnostic biopsies at a recently irradiated site is low (i.e. < 5%), if done within 2–4 weeks of starting radiation [2, 3]. In a study of the histologic remission of nasopharyngeal carcinoma (known to be a radiosensitive tumor), serial biopsies were taken weekly after a definitive course of radiation (median 61 Gy in 35–40 fractions). Fewer than 5% of patients had histologic remission 1 week after completion of radiation, corresponding to at least 8 weeks after radiation initiation [2]. In the Stockholm III trial for rectal cancer, the rates of pathologic complete response from surgery shortly (1–7 days) after short-course radiotherapy (RT) (25 Gy in 5 fractions) was 2.1%. Without any additional radiation, waiting 4–8 weeks after completion of radiation yielded a pathologic complete response rate of 11.8% [3]. In the MISSILE trial for early-stage non-small cell lung cancer (NSCLC) treated with stereotactic ablative radiotherapy (SABR), even 10 weeks after high-dose treatments, 40% of patients still had viable tumor cells by hematoxylin & eosin (H&E) staining [4].

Given these results, we postulate that the risk of a negative biopsy after radiation is likely overestimated by clinicians. In patients where a biopsy is obtained within weeks of palliative-dose radiation, we estimate the probability of a non-diagnostic biopsy as < 5%. The caveat is that though there is available data on the effects of radiation on histologic diagnosis, there is a lack of available data on the effects of radiation on molecular tests (i.e. EGFR, ALK, BRAF, etc.). This study will actively assess for the diagnostic quality of both immunohistochemical and molecular testing on biopsies of irradiated sites.

### Potential issue #2: increasing the risk of biopsy complications

Based on available literature, the risks of increased biopsy complications from a recently irradiated site are likely < 1% [5]. In a review of diagnostic biopsies performed in irradiated tissue, only 17 of 2160 patients were reported to have biopsy complications [5]. In this review, most studies did not actively evaluate for biopsy complications and relied on either chart review or patient reporting, as such, the study might underestimate the rates of low-grade complications. We will actively evaluate for biopsy complications of irradiated sites in our study.

### Potential issues #3 and 4: treating a non-malignant process or a malignancy in which upfront radiation would have been inappropriate

These two issues have historically been the main reasons for delaying radiation before the biopsy result is available, as they may either lead to unnecessary treatment and toxicity for a patient, or in the worst case, limit the ability to deliver curative-dose radiotherapy in the future (i.e. lymphoma). With modern imaging techniques (i.e. cross-sectional imaging and sometimes functional imaging), we believe clinicians are able to attain a high certainty of malignancy without a biopsy in certain settings. In these urgent palliative situations, there may be an inherent risk to delaying radiation, therefore physicians and patients should discuss the diagnostic probability of malignancy, the potential risks of upfront radiation as well as the possible consequences of delaying radiation. Ultimately, the physician and patient must come to a shared decision on whether upfront radiation is appropriate and in keeping with the patient’s clinical situation, preferences, and values. The data prospectively collected from this trial will hopefully better inform future physicians and patients on these risks.

## Methods

### Objective

To determine if it is safe and feasible to deliver palliative radiation before a biopsy result is available in a carefully selected group of patients with urgent indications for radiation.

### Study design

**Fig. 1:**
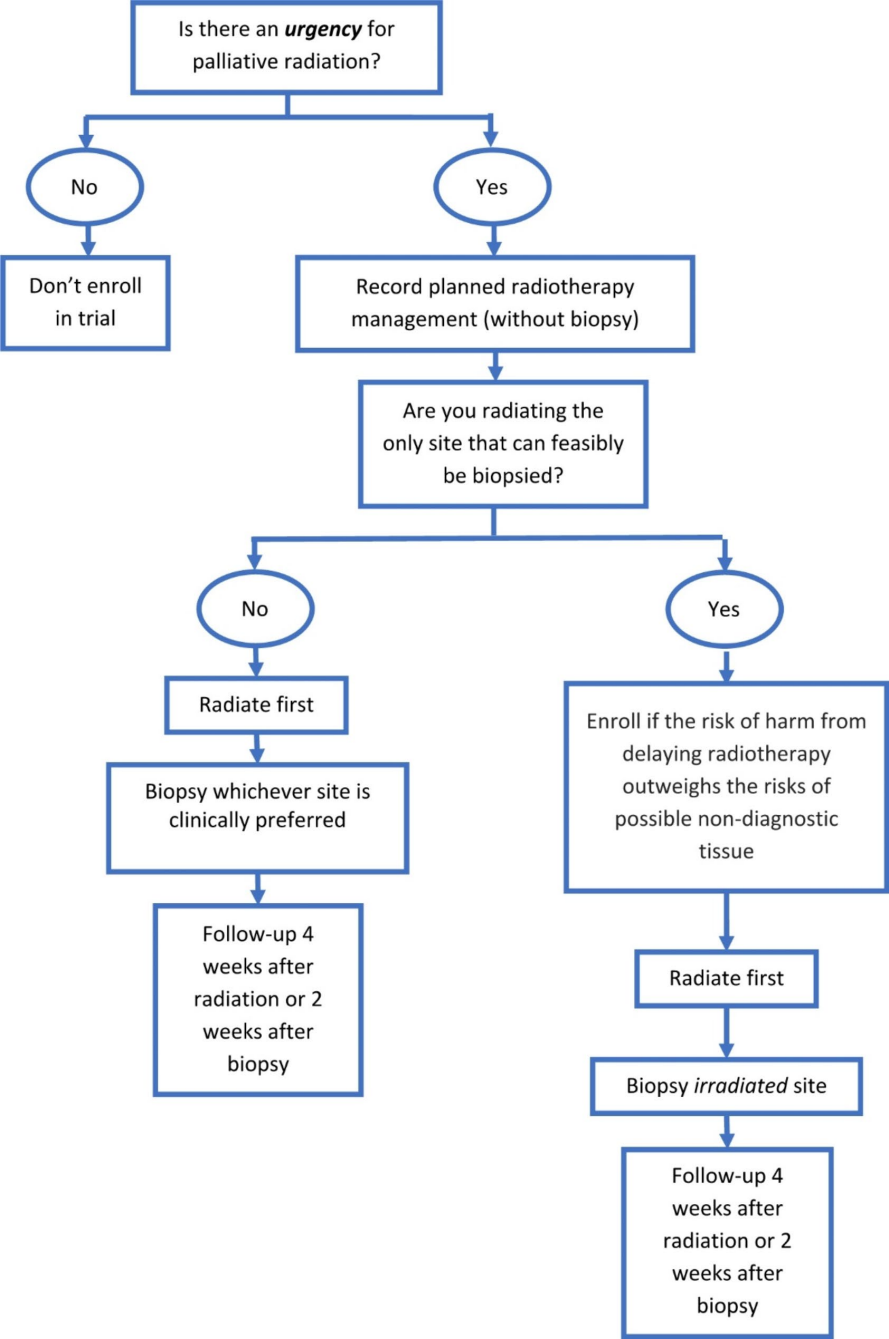
General study schema

This is a prospective, single arm pragmatic clinical trial assessing the feasibility of delivering palliative radiation before a biopsy result is available in patients with urgent indications for palliative radiation. Study schema is shown in Fig. 1. Participants will be enrolled by study personnel at each institution before delivery of radiation. Elements from PRECIS-2 were used to inform our pragmatic trial design [6, 7]. For example, we kept the eligibility broad across cancer types, with no required performance status scores, only one mandated follow up and no additional recruitment efforts beyond usual care.

We hypothesize that with a radiation-before-biopsy approach, the rate of “inappropriate radiation” will be 5% or less. We hypothesize that the biopsy yields to establish a histologic and molecular diagnosis will be acceptable and comparable to our institutional averages of 90–95% and 80–90%, respectively. We hypothesize that the rates of complications will be acceptable and comparable to our institutional average of 5–10%.

### Study endpoints

#### Primary endpoint

- Rate of “inappropriate use of radiation”, defined as the percentage of patients in whom management would have differed if the biopsy results were known prior to radiation. These cases meet the definition either when:
  - Biopsy pathology clearly shows a non-malignant entity.
  - Or the biopsy shows malignancy, but the patient would have received upfront systemic therapy or an alternate therapy, rather than radiotherapy, had the biopsy results been known.

#### Secondary endpoint

- Histological diagnostic accuracy, defined as the percentage of biopsies that yielded a histological diagnosis. This will include a comparison of diagnostic yield between biopsies done at radiated vs. non-radiated sites.
- Molecular testing accuracy, defined as the percentage of biopsies that yielded enough viable tissue for successful Next-Generation Sequencing testing using the ThermoFisher Oncomine V3 panel.
- Number of biopsy attempts required.
- Biopsy complication rates.
- Time from enrollment to first fraction of radiation.
- Time from enrollment to biopsy.
- Evidence of radiation effect in biopsy, defined as evidence of necrosis or dense fibrosis causing non-diagnosis in a lesion that had been radiated before the biopsy. If a biopsy was non-diagnostic because it was non-lesional, it would not be considered non-diagnostic as a result of radiation.
- Overall survival, defined as the time from enrollment to death from any cause or date of last follow-up, whichever occurs first.
- Edmonton Symptom Assessment Scale (ESAS) quality of life scores before RT and at first follow up (i.e. 4 weeks after RT).

### Patient selection

#### Inclusion criteria

- Age 18 years or older.
- Willing to provide informed consent.
- Palliative treatment intent: either metastatic or incurable locally advanced disease.
- Tissue diagnosis is not required for determination of dose/fractionation scheme.
- Recent cross-sectional imaging (e.g. computed tomography [CT], magnetic resonance imaging [MRI], positron emission tomography [PET]/CT) of the area to be treated, done within the past 3 months.
- Treating physician considers the pre-test probability of cancer > 95% based on clinical judgement and radiological findings.
- For patients with a prior history of malignancy, patients should be enrolled only when a biopsy is required for management decision-making. For example, these should be cases where urgent palliative radiation is delayed by the need for a biopsy. Patients can be enrolled, for example, under the following scenarios:
  - They have a prior malignancy but that prior malignancy is not considered the most likely histology for the current malignant lesion being treated.
  - Re-biopsy of the site being radiated is needed for molecular biomarkers.
- Radiation is considered urgent (i.e. patient should receive radiation prior to biopsy date). Urgent indications may include (Table 1):
  - Spinal cord compression (actual or impending) and inoperable.
  - Symptomatic brain metastasis and inoperable.
  - Other lesions causing neurologic deficit.
  - Pulmonary lesion causing or threatening lung obstruction.
  - Bleeding (including hemoptysis, upper/lower gastrointestinal bleed, hematuria).
  - Superior vena cava obstruction (actual or impending).
  - Cancer-related pain, not adequately responding to analgesia.
  - Impending pathologic fracture.
- The patient has at least 1 site of cancer amenable to biopsy.
- As per standard practices, if the radiation oncologist will be radiating the only site available to biopsy, they should proceed with caution. Patients should only be enrolled on trial if the risk of harm from delaying RT significantly outweighs the risks of possible non-diagnostic tissue. If the patient may potentially be eligible for systemic therapy, the treating radiation oncologist should consult a medical oncologist for an opinion regarding the risks of non-diagnostic molecular testing. The weighing of these priorities should be thoroughly discussed with the patient and the discussion should be documented. Reasons for radiating a patient with a single lesion prior to biopsy include:
  - Spinal cord compression (actual or impending) and inoperable.
  - Brain metastasis with significant symptoms or neurologic deficits and inoperable.
  - Other lesions causing neurologic deficit.
  - Pulmonary lesion causing or threatening lung obstruction.
  - Uncontrolled bleeding.
  - Superior vena cava obstruction (actual or impending).
  - Limited upside to molecular testing, as determined by the medical oncologist (i.e. patient unfit for available systemic therapies or limited options for systemic therapy).

**Table 1:** Examples of urgent indications for palliative radiotherapy

▪ Spinal cord compression (actual or impending) and inoperable
▪ Symptomatic brain metastasis and inoperable
▪ Other lesions causing neurologic deficit
▪ Pulmonary lesion causing or threatening lung obstruction
▪ Bleeding (including hemoptysis, upper/lower gastrointestinal bleed, hematuria)
▪ Superior vena cava obstruction (actual or impending)
▪ Cancer-related pain, not adequately responding to analgesia
▪ Impending pathologic fracture

#### Exclusion criteria

- Patient is potentially eligible for curative treatment.
- Patient has a tumour biomarker that clearly indicates the presence and type of cancer (e.g. highly elevated prostate-specific antigen [PSA]).
- Clinical suspicion of lymphoma > 20%.
  - Some features may be suggestive of lymphoma, including fever or night sweats (i.e. B symptoms excluding weight loss), or imaging showing well-defined, homogenous lymphadenopathy. These findings are not exclusion criteria specifically, but should be considered by the clinician in formulating their differential diagnosis.

### Pre-treatment evaluation

- History and focused physical examination (as needed).
- Eastern Cooperative Oncology Group (ECOG) performance status.
- Cross-sectional imaging (e.g. CT, MRI, PET/CT) within 3 months.
- Physician pre-treatment baseline questionnaire.
  - Treating physician must consider the pre-test probability of cancer as > 95% based on clinical judgement/radiological findings, and deem the probability of lymphoma < 20%.
  - Patient must not be considered potentially eligible for curative treatment.
- Radiation plan documented (dose/fractionation, site).
- ESAS quality of life score.

### Evaluation during treatment

No additional evaluations are required during treatment, apart from regularly scheduled visits with the treating physician. The treating physician should ask about any adverse events and biopsy complications during patient appointments, and document them in the patient’s chart as per institutional practice.

### Follow-up

In keeping with a pragmatic trial design [6], we will limit the number of additional follow-up visits beyond routine practice. We will do one follow-up either in person or over the telephone 4 weeks after RT or 2 weeks after biopsy, whichever is later. The purpose of this follow-up will be to assess for symptomatic response to radiation and monitor for adverse events from the biopsy. We will also obtain an ESAS score at the follow-up visit.

Post-treatment management questionnaires should be completed by the treating radiation oncologist (within 3 months of enrolment), which includes:

- Biopsy result, including histological and molecular diagnosis.
- Biopsy complications.
- Preferred management plan if biopsy results were known prior to enrolment/radiation.

In addition, we will review the participant’s chart 1 year after enrolment, to determine the survivorship status of the patient.

### Intervention

#### Radiation treatment

Radiation technique, volumes and dose/fractionation schemes will be decided by the treating radiation oncologist based on consideration of disease site, volume of tumour, nearby organs at risk, likely histology, performance status, patient age and prognosis. This allows a high flexibility of delivery, intended to keep the trial pragmatic and in keeping with real world practice [6]. It is not recommended to spare part of the tumor solely to allow for a biopsy.

#### Biopsy

Biopsies should be taken at whichever site is clinically preferred, typically the most anatomically feasible site of biopsy (regardless of whether it has been irradiated), considering institutional wait times and availability for different biopsy procedures. If the first biopsy attempt is unsuccessful, the second attempt can be a repeat biopsy at the same site or at another site.

#### Timing of biopsy and radiation

If the patient’s biopsy has not yet been ordered at the time of enrolment, it should be ordered immediately. The biopsy should be done at the earliest possible time, and if it is performed before radiation, or during radiation, the biopsy should proceed and the radiation should continue as planned. A biopsy may be completed before the patient is enrolled on trial as long as the biopsy result is not yet available at the time of enrolment (and at the start of radiation treatment).

### Adverse events

We will not be routinely evaluating for adverse events from radiation since these patients are receiving radiation regardless of enrolment (the trial is simply studying the *timing* of radiation in relation to biopsy).

However, we will actively evaluate for biopsy complications as it is possible that the frequency or severity of biopsy complications could increase if done within an irradiated area. In the follow-up visit post-treatment questionnaire, we will specifically evaluate for the following complications:

1. Pneumothorax (requiring a chest tube).
2. Wound infections (requiring antibiotics).
3. Prolonged bleeding from biopsy site.
4. Fistula formation.
5. Delayed wound healing, defined as non-healing wound or dehiscence at biopsy site > 6 weeks following the biopsy (requiring a specialist wound care team or continued antibiotics) – this should be assessed from the chart and included in post-treatment questionnaire.

### Statistics and sample size calculations

We hypothesize that the primary endpoint, rate of “inappropriate radiation”, will be no more than 5%. We consider it unacceptable if the rate of “inappropriate radiation” is ≥ 20%. Using a one-sided, one-sample binomial test, with 90% power at the 5% significance level, to reject the null hypothesis of a change rate of ≥ 20%, with 10% drop-out, we estimate that 48 patients are needed.

### Accrual/study duration considerations

We estimate accrual of 2–4 patients per month. As such, the study is projected to take between 1 and 2 years to accrue, with an additional 3 months required for follow-up. Patients who are eligible will be recruited in usual care settings by physicians. No additional promotional material will be made to recruit patients.

### Planned safety interim analysis

One interim analysis will be done once half of the patients (*n* = 24) have been accrued and their data are fully collected. The trial will be stopped if the rate of “inappropriate radiation” is ≥ 20% (*n* ≥ 5). Additionally, in the subset of patients where there is only 1 feasible site of biopsy and that site was irradiated, if the yield of histological diagnosis is < 80% or yield of molecular testing is < 70%, then no further patients will be enrolled where the site of radiation is the site of biopsy.

### Data analysis

Patients who decline a biopsy after RT will be excluded from analysis of both primary and secondary endpoints. Patients for whom biopsy is repeatedly non-diagnostic after undergoing RT will be masked from analysis of the primary endpoint, since inappropriate use of RT cannot be determined without a biopsy. However, analysis of secondary endpoints will be conducted, including determination of overall rates of diagnostic yields/accuracy, complications from biopsy, and number of biopsy attempts.

#### Subject discontinuation and withdrawal

Subjects may voluntarily withdraw from study participation at any time. If a subject is removed from the study, an attempt should be made to obtain the follow-up evaluation that would have been obtained at the completion of the study, provided the patient consents to do so.

#### Risks to subjects

The risks of participating in the study are outlined in the introduction as inherent risks of delivering radiation without a pathologic diagnosis:

1. Creating a non-diagnostic biopsy through radiation effects on tissue.
2. Possible increase in the risk of biopsy complications.
3. Risk of treating a non-cancerous process.
4. Risk of treating a cancer that would be best managed with chemotherapy/systemic therapy as the primary treatment (i.e. lymphoma, small cell lung cancer, germ cell tumor).

We will be measuring each of these risks carefully during the study to determine whether the level of risk is acceptable as a standard of care approach in this selected population. In our population, there is also a risk of *not* participating in the study: the cancer may progress and cause symptoms and complications during the waiting period for a biopsy sample.

#### Risk to subjects undergoing radiation to the only available site of biopsy

For the subset of patients with a single lesion to biopsy, the reason for urgent radiation treatment must be carefully documented and risks of non-diagnostic biopsy discussed with the patient. Any medical oncologist assigned to the patient should be consulted to determine the potential negative impacts if molecular testing is not able to be obtained. If the patient does not have an assigned medical oncologist, the treating radiation oncologist can consider reaching out to a medical oncologist specific to that disease site or the medical oncologist on call, but this is not absolutely required. There will be a safety interim analysis conducted as discussed under the planned safety interim analysis section, and if the yield of histological diagnosis is < 80% or yield of molecular testing is < 70%, then no further patients will be enrolled where the site of radiation is the site of biopsy.

#### Confidentiality of subject records and data storage

All data will be stored on REDCap, which is a secure web application for building and managing online databases commonly used in the clinical trials research community.

#### Financial support for patients

Patients are not financially renumerated on this trial.

## Discussion

This pragmatic single-arm clinical trial seeks to collect prospective data on patients with presumed metastatic (or otherwise incurable) cancer where there is an urgent need for palliative radiation. There is currently a paucity of data to guide empiric decision-making for radiation oncologists and patients in these situations. By treating all such patients with upfront palliative radiation on trial before biopsy results are available, we will prospectively measure the accuracy of modern imaging and clinical acumen in guiding radiotherapy decision-making in the palliative oncology setting. We believe that if the rate of inappropriate radiation is found to be no more than 5%, then this approach should be considered safe and feasible in the palliative setting.

**Fig. 2:**
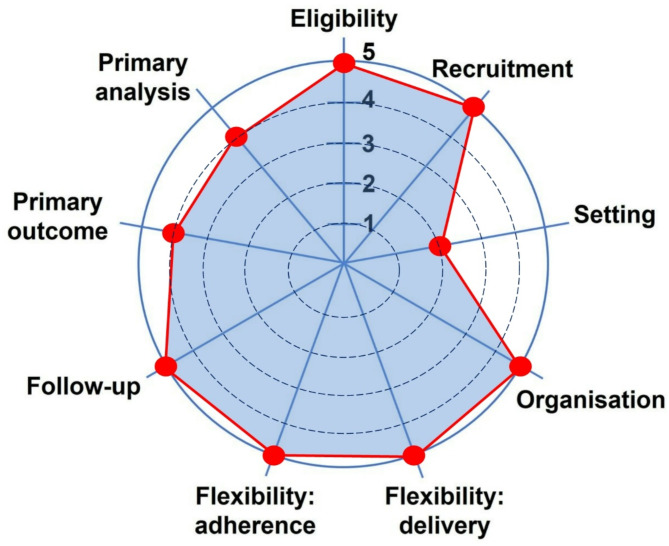
PRECIS-2 wheel for RT-NOW trial

RT-NOW was designed pragmatically to mimic real world practice [6] and the PRECIS-2 tool was used for trial design considerations (Fig. 2). For instance, we allowed broad eligibility across patients with different cancer histologies, performance statuses and radiation indications. Limited inclusion/exclusion criteria allowed treating physicians to consider the nuances specific to each patient, scenario and cancer type. We only requested that physicians need to be quite certain (> 95% likelihood) they are treating an incurable malignancy (based on available investigations) and that the risks of delaying radiation outweigh the risks of upfront radiation. There were no recruitment efforts beyond usual care. Radiation treatment schedules and techniques were decided by the treating physician, again reflecting our pragmatic approach. In addition, the limited number and scope of study follow-up visits was intended to mimic real-life practice in the palliative settings and the burden on patients and their caregivers. These patients often have limited life expectancies, and we do not want to overburden them with additional appointments. In addition, we chose the validated ESAS questionnaire to assess patient’s symptom burden and quality of life, given its ease-of-use, brevity and ability to add additional symptoms most specific to the patient [8].

One of the main risks of this study is the downstream impact a non-diagnostic biopsy may have on available systemic therapy options for the patient, especially in the era of targeted agents and immunotherapy. For example, it would be a significant error if pre-emptive radiotherapy to the biopsy site led to a non-diagnostic result in a patient that would have been eligible for life-prolonging targeted therapy. In patients with several sites feasible to biopsy, these risks are minor, as an unirradiated site may be biopsied even if the initial biopsy of an irradiated site was non-diagnostic. In that situation, the only harms would be the inconvenience and risks associated with an additional biopsy procedure. However, in patients with only one feasible site to biopsy, if radiation was to render the biopsies non-diagnostic, a successful re-biopsy may only be possible at disease progression. Given the risks unique to this set of patients, we will collect data on biopsy yields prospectively and do an interim safety analysis once half the patients have been accrued and their full data are collected.

To our knowledge, there are currently no similar trials studying this question of upfront palliative radiation in patients without a biopsy. Prospective data from this trial will be valuable in helping to guide the complex decision-making of physicians and cancer patients in these urgent palliative situations.

## Conclusions

RT-NOW is a pragmatic single-arm trial studying patients who are believed to have incurable cancers, where a biopsy is delaying urgent palliative radiation. The aim of this trial is to prospectively collect data on a radiation-before-biopsy approach to determine the rates of inappropriate management, biopsy complications and non-diagnostic biopsies. Such evidence is critically needed to help oncologists and patients make informed management decisions in these urgent situations.

## Data Availability

No datasets were generated or analysed during the current study.

## Declarations

### Data safety monitoring board

This study does not require a data safety monitoring board.
